# Determinants of Exclusive Breastfeeding in South Gujarat Region of India

**DOI:** 10.4021/jocmr2009.06.1242

**Published:** 2009-06-21

**Authors:** Rajesh K Chudasama, Panna C Patel, Abhay B Kavishwar

**Affiliations:** a Department of Community Medicine, P D U Medical College, Rajkot, India; b Department of Pediatrics, Government Medical College, Surat, India; c Department of Community Medicine, Government Medical College, Surat, India

## Abstract

**Objective:**

To estimate the situation of breastfeeding in Surat among infants and to determine variables associated to major risks for early weaning.

**Design and settings:**

Mothers coming to the well baby clinic for immunization of infants at Government Medical College and Hospital were interviewed using pretested questionnaire.

**Subjects:**

Mothers with their infants who have not completed one year of age.

**Methods:**

In this cross sectional study, 498 mothers were selected for study from May to September, 2008. Survival analysis was the method used to calculate the prevalence and the median duration of breastfeeding. The Chi-square test was performed to compare the proportions; significance level was set at 5%. Odds ratio was used to measure the significance of association, with a 95% confidence interval. Logistic regression analysis was used to identify the risk factors for early weaning.

**Results:**

The median length of exclusive breastfeeding was 6 months. Risk factors for early weaning were primiparity (OR = 3.01, 95% CI = 2.01- 4.51), consecutive delivery interval less than 24 months (OR = 1.79, 95% CI = 1.09 - 2.92), maternal age below 20 years (OR = 6.49, 95% CI = 2.69 - 15.61), and paternal occupation as labor (OR = 2.02, 95% CI = 1.36 - 3.00).

**Conclusions:**

Exclusive breastfeeding practices are not in a better situation than at national level. The factors related to early weaning denote a weak breastfeeding support given by maternal and infant health services.

**Keywords:**

Exclusive breastfeeding; Weaning; Antenatal care; Postnatal care; Education

## Introduction

Mothers milk undoubtedly represents the best nourishment for the child during first months of life. There is a universal consensus about the fundamental importance for childrens adequate growth and development and for their physical and mental health. The benefits of breastfeeding (BF) specially, exclusive breastfeeding (EBF), are well established [1, 2] particularly in poor environments where early introduction of other milk is of particular concern because of the risk of pathogens contamination and over dilution of milk leading to increased risks of morbidity and undernutrition [1] . No artificial feeding formula is capable of qualitatively replacing breast milk, its specific nutrients and protection against diseases [3]. All women should be enabled to practice exclusive breastfeeding and all infants should be fed exclusively on breast milk from birth to 4 to 6 months of age and thereafter, children should continue to be breastfed, while receiving appropriate and adequate complementary foods, for up to 2 years of age or beyond [4]. Too early introduction of breast milk substitutes and too late introduction of semi solid complementary feeds are common and are responsible for rapid increase in the prevalence of under nutrition between 6-24 months [5]. Breastfeeding is promoted internationally as the preferred method of feeding infants up to 6 months and continued up to two years with the addition of home cooked food [6, 7].

Exclusive breastfeeding defined by World Health Organization (WHO) as practice of feeding only breast milk (including expressed breast milk) and allows the baby to receive vitamins, minerals or medicines and water, breast milk substitutes, other liquids and solid foods are excluded. World Health Assembly of WHO in 2001 made resolution that exclusive breastfeeding for the first six months is the most appropriate infant feeding practice [8]. Recent developments suggest full breastfeeding should continue to six months and there is good evidence that two more months of EBF from fourth to six months provides infants with additional protection against gastrointestinal and acute respiratory infections during that two months period [9]. In recent decades, breastfeeding promotion, protection and support actions have been implemented as a strategy to reduce child mortality and improve childrens health.

The present study aimed at establishing knowledge about the reality of breastfeeding in the urban area of South Gujarat region. We also aimed at identifying the demographic and socioeconomic variables related to maternal and infant assistance that may be negatively interfering in breastfeeding practices.

## Methods

This study was done in urban areas of Surat in South Gujarat region from May, 2008 to September, 2008. The study participants were mothers with their infants from 0-11 months age. Total 498 mothers with their infants attending well baby clinic for immunization were interviewed at Government Medical College and Hospital, Surat during above mentioned period. Every third mother with her infant attending the well baby clinic was included for study. We considered as losses the mothers who refused to participate in the study (total 18 mothers). The sample size calculated was 478 infants, using EPI 6 software with 46% children exclusive breastfed up to 6 months [10], standard error of 5% and design effect 5. The study was planned with purposive sampling, in which every third mother was included from the beginning of study period and on achieving the calculated sample size, data collection was terminated. Informed consent was obtained from mothers who agreed to participate in study. Limitation of present study was selection bias as it was a hospital based study. Women recruited for study were from homogenous group and their sociodemographic variables did not differ from those who did not participate in the study.

During interview, we used forms with direct, easy to answer questions and did not open many answering possibilities, requiring short answers. Questions included information on demographic and socio-economic variables of the mothers and on local maternal and infant assistance in addition to childrens eating habits. The studied variables were divided into following groups. (1) Socio-demographic variables. Parity, inter delivery interval, mode of delivery, birth weight, maternal age, type of family (nuclear or joint), socio-economic status, maternal education, paternal education, paternal occupation (labor or others). (2) Variables related to antenatal care (ANC) and postnatal care (PNC). Number of antenatal visits, received advice regarding breastfeeding during antenatal period and during postnatal visit, when started breastfeeding after birth, type of feeding - on demand or at fixed time, any initial difficulties during breastfeeding, previous duration of exclusive breastfeeding in multiparous women.

The Epi Info software (version 3.5.1) [11] was used for processing and analyzing data. The Chi-square test was applied for comparing proportions when we evaluated factors associated with early weaning: a 5% alpha error was admitted. We also calculated odds ratio for each studied variable, with a 95% confidence interval. This is usually applied in crossover studies, in which prevalence instead of incidence is assessed according to the significance of association. Logistic regression was performed with same software, to identify variables that in a simultaneous mathematical analysis presented an independent explanatory effect on higher risk for early weaning. For this stage of investigation, we selected the variables that showed P < 0.20 in the bivariate analysis. Survival analysis was used to calculate the prevalence and the median duration of breastfeeding.

Breastfeeding defined here into following categories, was proposed by World Health Organization [12]: exclusive breastfeeding as when child is fed exclusively on human milk; predominant breastfeeding when child is fed on human milk and other liquids like water, tea, juices; general breastfeeding when all kind of milk, liquid and semisolid diet is given. Early weaning in this text refers to introduction of complementary feeding prior to 6 months of life, with interruption of exclusive or predominant breastfeeding before this period.

## Results

In our observed group, 55% infants were male while 45% were female. As shown in Table 1, majority of interviewed mothers were multiparous (67.5%), and the time interval between two deliveries was more than 24 months in 73.5% mothers. Large majority (89%) of infants were born by normal deliveries. Among all the delivered babies, 44.5% infants had low birth weight (< 2,500 grams). The proportion of mothers under 20 years of age was 9%. Two third families (66%) were nuclear and 40% study participants belonged to lower socio-economic class. Among participated mothers, 27.7% were illiterate, 35% mothers had education up to primary level and remaining 37% had higher education, while only 10% fathers were illiterate. Labor work (71.5%) was main profession of fathers and remaining was engaged in other works, like shop keeping, business, and service.

**Table 1:** Bivariate analysis of socio-demographic variables and association with early weaning

Variable	Weaning		χ^2^	P	RR*	CI 95%
	Yes (n = 229)	No (n = 269)				
Parity						
Primiparous	46	116	29.9	0.001	2.14	1.60 - 2.87
Multiparous	183	153								
Inter-delivery interval						
< 24 months	39	50	5.53	0.018	1.34	1.06 - 1.70
≥ 24 months	144	103								
Mode of delivery						
Operative	31	22	3.73	0.053	1.33	0.96 - 1.86
Normal	198	247								
Birth weight						
< 2500 gms	97	125	0.84	0.357	1.07	0.91 - 1.26
≥ 2500 gms	132	144								
Maternal age						
< 20 years	6	40	22.14	0.001	1.71	1.48 - 1.98
≥ 20 years	223	229								
Type of family						
Nuclear	148	181	0.38	0.532	1.05	0.88 - 1.25
Joint	81	88								
Socio economic status						
Upper & Middle	165	134	25.49	0.001	0.66	0.56 - 0.77
Lower	64	135								
Maternal education						
Illiterate	55	83	2.88	0.089	1.16	0.98 - 1.37
Literate	174	186								
Paternal education						
Illiterate	37	13	17.56	0.001	0.45	0.28 - 0.73
Literate	192	256								
Paternal occupation						
Labor	146	210	12.42	0.001	1.41	1.14 - 1.75
Others	83	59								

*RR: Risk Ratio

**Figure 1:**
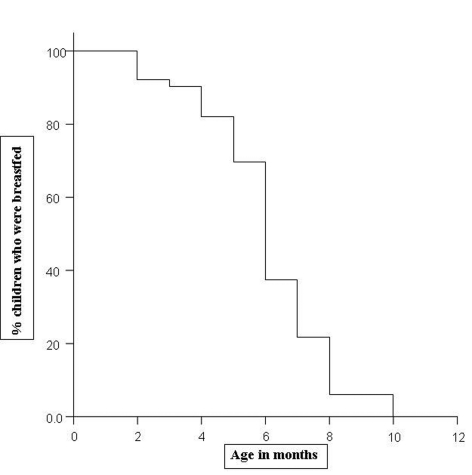
Survival curve showing survival probability for duration of exclusive breastfeeding

On the basis of life table technique, Figure 1 shows survival probability for duration of exclusive breastfeeding among study infants. The life table technique allows us to consider children who are still being breastfed at the time of interview of mothers, and also to know the proportion of children that remain being breastfed by the end of each month of their life. So, it allows a longitudinal approach to the cross sectional data collected. In relation to exclusive breastfeeding, we observed that this was a universal practice immediately after birth and it continues for first three months of life. By the end of fourth month, more than 80% children were still exclusively breastfed, which further drops to 70% by the end of the fifth month. At the end of 6 months, only 37% children were exclusively breastfed. The median length of exclusive breastfeeding (age in which half of the children received only the mothers milk) was found 6 months (with 95% confidence interval 5.81 6.189) in present study.

Table 2 shows studied variables related to antenatal and postnatal care and their association with early weaning. Three antenatal visits were taken by 77% mothers, and 86% mothers received advice regarding breastfeeding during their antenatal visits. Almost half (45.4%) of the mothers started breastfeeding within first hour after delivery, 39% started within first six hours, while only 15.5% started after 6 hours of delivery. Only 18% mothers reported some initial difficulty in breastfeeding. Among multiparous women, 75% had given exclusive breastfeeding to their child for 6 months or more.

**Table 2:** Bivariate analysis of variables related to antenatal care (ANC), postnatal care (PNC) and their association with early weaning

Variable	Weaning		χ^2^	P	RR*	CI 95%
	Yes (n=229)	No (n=269)				
Antenatal care (ANC)						
< 3 visits	56	57	0.75	0.386	1.09	0.89 - 1.33
≥3 visits	173	212								
BF advice during ANC						
Received	196	235	0.33	0.563	1.07	0.83 - 1.38
Not received	33	34								
First BF started within						
< 6 hrs	200	221	2.53	0.111	0.84	0.69 - 1.02
> 6 hrs	29	48								
Initial difficulties						
Yes	50	41	3.59	0.057	1.24	0.97 - 1.58
No	179	228								
Type of feeding						
On demand	177	215	0.51	0.474	1.07	0.87 - 1.32
Fixed time	52	54								
BF advice during PNC						
Received	202	244	0.82	0.363	1.13	0.73 - 2.31
Not received	27	25								
Previous EBF duration in multiparous						
< 6 months	46	36	0.11	0.732	0.95	0.72 - 1.25
≥ 6 months	137	117								

*RR: Risk Ratio

Among variables chosen for the logistic regression (Table 3), primiparity (OR = 3.01, 95% CI = 2.01 - 4.51), interval between two deliveries less than 24 months (OR = 1.79, 95% CI = 1.09 - 2.92), maternal age less than 20 years (OR = 6.49, 95% CI = 2.69 - 15.61), and paternal occupation as labor (OR = 2.02, 95% CI = 1.36 - 3.00) were the factors associated with higher risks for early weaning.

**Table 3:** Logistic regression analysis for variables related to higher risk for early weaning

Variable	Coefficient (β)	Standard deviation	Odds ratio (OR)	CI 95%
Parity	1.10	0.2058	3.01*	2.01 - 4.51
Inter-delivery interval	0.58	0.2496	1.79*	1.09 - 2.92
Mode of delivery	0.56	0.29	1.75	0.98 - 3.13
Maternal age	1.87	0.44	6.49*	2.69 - 15.61
Social class	-0.95	0.19	0.38*	0.26 - 0.56
Maternal education	0.34	0.20	1.41	0.94 - 2.10
Paternal education	-1.33	0.33	0.26*	0.13 - 0.50
Paternal occupation	0.70	0.20	2.02*	1.36 - 3.00
First BF started late	-0.40	0.25	0.66	0.40 - 1.09
Initial difficulties	0.44	0.23	1.55	0.98 - 2.45

*P < 0.05

## Discussion

Exclusive breastfeeding is safe, economical and emotionally satisfying means of feeding babies, particularly in developing country like India and probably for others. In countries where lactation support is available, six months exclusive breastfeeding has improved substantially over the time [8].

This study enabled to evaluate the rate of exclusive breastfeeding and to determine factors associated with cessation of exclusive breastfeeding within first 6 months of life. The study showed that all the mothers had started breastfeeding after delivery. Prevalence rate of exclusive breastfeeding by 6 months was 54%, little higher than national level (46%) as reported by National Family Health Survey 3 (NFHS 3) [10]. Median length of exclusive breastfeeding was 6 months, which was also reported in another study from same geographic area [13]. Giashuddin MS et al [14] and Caldeira AP et al [15] have reported median duration of EBF as 3.67 months and 1 month respectively, which is lower than the present study.

In relation to variables involved in breastfeeding, this study assessed the factors associated with a higher risk for early weaning, here it be understood as the introduction of foods other than mothers milk before a child attains 6 months of age. Generally, socio-demographic variables are not very likely to be relevant to the breastfeeding pattern. A review performed by Losch et al [16] pointed out several works with different results in relation to these variables. The present study showed primiparity, delivery interval more than 24 months, early maternal age, lower socio-economic status, low paternal education and occupation as a risk factor for early weaning, even after the logistic regression. Authors have reported positive association for early maternal age and lower socio-economic status in their previous study also [13]. Agampodi SB et al [17] have reported influence of paternal education and maternal employment in their study, and no association was found for variables like, low birth weight, type of family and maternal education. In contrast, others have reported low birth weight [15] and illiteracy or lower maternal education [18] as high risk factor for early weaning in their study.

Bivariate analysis of Table 2 shows the lack of association between exclusive breastfeeding and variables classically considered as supportive of breastfeeding like, number of antenatal visits, advice received regarding breastfeeding during antenatal period and during postnatal period, initiation of breastfeeding within one or first few hours, and type of feeding whether on demand or at fixed time as also reported by Singh G [19] in his study. The possible justification for such findings could be excellent execution of maternal and infant care which includes promotion of breastfeeding in health services especially after introduction of Integrated Management of Neonatal and Childhood Illnesses (IMNCI) training which promotes exclusive breastfeeding for first 6 months of life. Breastfeeding is a maternal option that involves a complex interaction of socioeconomic, cultural and psychological factors and many more. However, as a socially recreated habit, the role of reproductive and child health services in promoting of breastfeeding should by no means be disregarded.

The main reasons presented by mothers as difficulties in initiating breastfeeding were infant not sucking effectively (80%), retracted nipple (53%), cracked nipple (28%) or milk was absent (20%) for first few days after delivery. Some authors have reported that main reasons for difficulties in initiating breastfeeding were belief that just mothers milk was not sufficient, excessive crying, little milk, thinned milk [15]. As the study was hospital based, it introduces some selection bias, and because the data collection was based on mothers recall, it also introduces recall bias and these were the limitations of present study.

In conclusion, exclusive breastfeeding practices in study group are not in a better situation than at national level. The factors related to early weaning denote a weak breastfeeding support given by maternal and infant health services.
